# Ceftriaxone-Resistant *Neisseria gonorrhoeae*, Japan

**DOI:** 10.3201/eid1701.100397

**Published:** 2011-01

**Authors:** Makoto Ohnishi, Takeshi Saika, Shinji Hoshina, Kazuhiro Iwasaku, Shu-ichi Nakayama, Haruo Watanabe, Jo Kitawaki

**Affiliations:** Author affiliations: National Institute of Infectious Diseases, Tokyo, Japan (M. Ohnishi, S. Nakayama, H. Watanabe);; Mitsubishi Chemical Medience Corporation, Tokyo (T. Saika); Hoshina Clinic, Kyoto, Japan (S. Hoshina);; Kyoto Prefectural University of Medicine, Kyoto (K. Iwasaku, J. Kitawaki)

**Keywords:** Neisseria gonorrhoeae, antimicrobial resistance, sexually transmitted diseases, STD, ceftriaxone, sex workers, gonorrhea, bacteria, Japan, letter

**To the Editor:** Spread of multidrug-resistant *Neisseria gonorrhoeae* is a major public health concern. Effective antimicrobial therapy is a key element in gonorrhea control. However, *N. gonorrhoeae* has developed resistance to multiple classes of antimicrobial drugs, including β-lactams, tetracyclines, and fluoroquinolones (*1**–**3*). Even an extended-spectrum oral cephalosporin-resistant, cefixime-resistant *N. gonorrhoeae* has emerged, and cefixime has now been withdrawn from use in Japan. Best practice treatment is limited to injectable extended-spectrum cephalosporins, such as ceftriaxone and spectinomycin. The emergence of ceftriaxone-resistant *N. gonorrhoeae* threatens effective disease control.

We identified a novel ceftriaxone-resistant *N. gonorrhoeae* isolated from a 31-year-old female commercial sex worker; MIC of ceftriaxone for this isolate was high (2 µg/mL). The woman visited a clinic in Kyoto for a routine examination for sexually transmitted infections in January 2009. Although she had no obvious symptoms or signs, a throat sample collected on her first visit yielded a positive result for *N. gonorrhoeae* by the strand displacement amplification test (ProbeTec ET, Becton Dickinson, Franklin Lakes, NJ, USA), but a vaginal sample taken at the same time was negative. After 2 weeks, another throat sample was positive for *N. gonorrhoeae* when cultured on Thayer-Martin medium, and the patient subsequently received 1 g ceftriaxone intravenously. Her pharyngeal sample was also *N. gonorrhoeae* positive by strand displacement amplification test on the third visit 2 weeks later, and further ceftriaxone treatment was prescribed. However, a culture for test of cure was not conducted because reinfection was considered. A negative result was finally obtained in April 2009.

The culture showed positive reactions in oxidase and catalase tests. Gram staining showed gram-negative diplococci. The ID-test HN-20 Rapid system (Nissui, Tokyo, Japan) classified the bacterium as *N. gonorrhoeae*. Susceptibility was determined by the agar dilution method (*4*). For this strain, named H041, MIC of ceftriaxone was high (2 µg/mL), and the strain was highly resistant to penicillin G (4 µg/mL), cefixime (8 µg/mL), and levofloxacin (32 µg/mL). However, it demonstrated susceptibility to spectinomycin (16 µg/mL) and reduced susceptibility to azithromycin (0.5 µg/mL).

To characterize the ceftriaxone-resistant *N. gonorrhoeae* H041, multilocus sequence typing characterized the strain as ST7363 (*5*), which is the predominant sequence type (ST) among cefixime-resistant clones (*6*). *N. gonorrhoea* multiantigen sequence typing (NG-MAST) was also performed (*7*). The NG-MAST strategy uses 2 genes, *por* and *tbpB*, for porin and a transferrin-binding protein, respectively. NG-MAST indicated that the strain H041 was ST4220 and contained the *por2594* allele and the *tbpB10* allele. NG-MAST 4220 is a novel ST. However, the *tbpB10* allele is the most frequently observed allele (76.5%) among multilocus sequence typing-ST7363 *N. gonorrhoeae* strains (n = 81) (M. Ohnishi, unpub. data).

Molecular typing suggested that the novel ceftriaxone-resistant *N. gonorrhoeae*, H041, is closely related to the ST7363 cefixime-resistant *N. gonorrhoeae*. Therefore, we compared *Spe*I-digested genomic DNA banding patterns of strain H041 with those of other *N. gonorrhoeae* strains by using pulsed-field gel electrophoresis as described (*8*). Four ST7363 strains, including *N. gonorrhoeae* H041, and 4 ST1901 strains (another major ST among cefixime-resistant *N. gonorrhoeae* strains) (*6*) were analyzed. The banding pattern of *Spe*I digested H041 genomic DNA was similar to that of other ST7363 strains and indistinguishable from that of cefixime-resistant but ceftriaxone-susceptible NG0207 (Figure).

**Figure:**
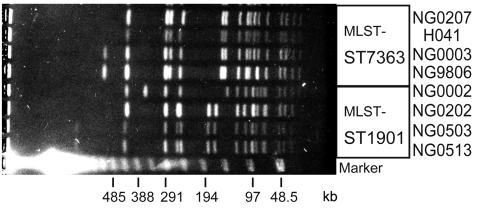
Pulsed-field gel electrophoresis patterns of ceftriaxone-resistant *Neisseria gonorrhoeae* strain H041 and other multilocus sequence typing (MLST) ST7363 and ST1901 strains. *Spe*I-digested genomic DNA from ceftriaxone-resistant *N. gonorrhoeae* H041, 3 of the MLST ST7363 strains and 4 of the MLST ST1901 strains were analyzed by pulsed-field gel electrophoresis. A lambda ladder standard (Bio-Rad, Hercules, CA, USA) was used as a molecular size marker.

We describe the emergence of ceftriaxone-resistant *N. gonorrhoeae*, isolated from a pharyngeal specimen from a female commercial sex worker. At 2 µg/mL, the MIC was 4-fold higher than that of the previously reported ceftriaxone nonsusceptible strain (*9*). Our susceptibility testing suggests that only azithromycin and spectinomycin are effective drugs for treating this strain. In this case, eradication was successful, although *N. gonorrhoeae* colonization of the pharynx may just be tempory because the pharynx is not an ideal site for *N. gonorrhoeae* growth. From the routine examinations of commercial sex workers during January–March 2009, 40 *N. gonorrhoeae* were isolated in the clinic, but no other ceftriaxone-resistant strains were isolated. There is no evidence of dissemination of this strain in Kyoto.

Three independent molecular subtyping methods indicated that the ceftriaxone-resistant H041 strain was *N. gonorrhoeae*, and it might originate from an ST7363 cefixime-resistant *N. gonorrhoeae* clone. There are several possible mechanisms for the acquisition of resistance, including formation of a new mosaic type *penA* allele as *penA-X* cefixime resistance and acquisition of an extended-spectrum β-lactamase gene. The H041 strain did not produce β-lactamase in a nitrocephin test. Further molecular analysis is needed to elucidate the precise mechanism of the ceftriaxone resistance of the H041 strain.

The emergence of ceftriaxone-resistant *N*. *gonorrhoeae* raises concerns for controlling gonorrhea because ceftriaxone is widely recommended and the first-line treatment for gonorrhea around the world. *N. gonorrhoeae* has a potential to gain an extraordinarily high MIC to ceftriaxone. Surveillance for ceftriaxone-resistant *N*. *gonorrhoeae* should be strengthened.
